# An Improved Experiment for Measuring Lithium Concentration-Dependent Material Properties of Graphite Composite Electrodes

**DOI:** 10.3390/nano12244448

**Published:** 2022-12-14

**Authors:** Huibing Liu, Guoxing Zhang, Dawei Li, Junqian Zhang

**Affiliations:** 1 Shanghai Institute of Applied Mathematics and Mechanics, School of Mechanics and Engineering Science, Shanghai University, Shanghai 200444, China; 2 School of Mechanical Engineering, University of Shanghai for Science and Technology, Shanghai 200093, China; 3 Zhejiang Laboratory, Hangzhou 311100, China

**Keywords:** graphite composite electrode, real-time and simultaneous quantification, partial molar volume, elastic modulus, diffusion induced stress

## Abstract

The in situ curvature measurement of bilayer beam electrodes is widely used to measure the lithium concentration-dependent material properties of lithium-ion battery electrodes, and further understand the mechano–electrochemical coupling behaviors during electrochemical cycling. The application of this method relies on the basic assumption that lithium is uniformly distributed along the length and thickness of the curved active composite layer. However, when the electrode undergoes large bending deformation, the distribution of lithium concentration in the electrolyte and active composite layer challenges the reliability of the experimental measurements. In this paper, an improved experiment for simultaneously measuring the partial molar volume and the elastic modulus of the graphite composite electrode is proposed. The distance between the two electrodes in the optical electrochemical cell is designed and graphite composite electrodes with four different thickness ratios are measured. The quantitative experimental data indicate that the improved experiment can better satisfy the basic assumptions. The partial molar volume and the elastic modulus of the graphite composite electrode evolve nonlinearly with the increase of lithium concentration, which are related to the phase transition of graphite and also affected by the other components in the composite active layer. This improved experiment is valuable for the reliable characterization of the Li concentration-dependent material properties in commercial electrodes, and developing next-generation lithium batteries with more stable structures and longer lifetimes.

## 1. Introduction

Lithium-ion batteries (LIBs) have the advantages of light weight and high energy density; they have been selected as the premier energy storage device for electric vehicles and portable electronic devices [1,2,3]. The intercalation/extraction of lithium (Li) ions induces expansion/contraction deformation of the active composite layer, and the constraint of the current collector and the battery case induce mechanical stress in the electrodes [4,5,6,7,8]. This diffusion-induced stress (DIS) can cause failures of active materials and electrode structures, and thus affect the cycling performance and stability of LIBs [9,10,11,12,13]. Therefore, measuring the Li concentration-dependent material properties of electrodes is essential to evaluating the DIS of electrodes, analyzing mechanical failures, and understanding the mechano–electrochemical coupling behavior of LIBs.

Several theoretical methods have been explored to analyze the DIS and calculate the material properties of electrodes. Zhang et al. obtained an analytical formula for the DIS of a layered electrode plate and evaluated the effects of structure and material parameters on the DIS [14]. Cheng and Verbrugge developed analytical expressions for the evolution of stress and strain energy in a spherically shaped electrode element under either galvanostatic or potentiostatic conditions [15]. Song et al. obtained an analytical solution for the diffusion stress during the lithiation of multilayer cylindrical LIBs and provided insights into the design of LIBs by analyzing the relationship between DIS and geometric parameters, material properties, and charging procedures [16]. Graphite, with excellent cycling stability, is widely used in anodes for commercial LIBs [17,18,19]. For this reason, computational [20,21] and experimental [22] studies on graphite and graphite composite electrodes have been investigated in recent years. Based on density functional theory (DFT), Qi et al. studied the polycrystalline Young’s modulus of graphite triples as it is lithiated to LiC6; the lattice and volume expansion increases with Li concentration [23]. Due to the lack of experimental data, the elastic modulus and volume expansion of the electrodes in the theoretical models was assumed to be linearly related to the Li concentration. Presently, numerous in situ experimental methods have been developed, such as X-ray diffraction (XRD), digital image correlation (DIC), and optical sensor [24,25,26,27]. For example, Yao et al. quantified the Li concentration gradient in graphite electrodes along the thickness direction using *operando* energy dispersive XRD [28]. Qi et al. combined DIC to describe direct in situ deformation-strain maps during lithium insertion and extraction in a composite lithium battery electrode [29]. Blanquer et al. developed an optical sensor for *operando* stress monitoring in lithium-based batteries, the optical signal was monitored during battery cycling, further translated into stress, and correlated with the voltage profile [30]. Although the electrochemical and mechanical responses during electrochemical processes could be obtained by direct measurement techniques, Li concentration-dependent material properties still need to be determined, which can provide essential insights into the working mechanism of the battery.

The Li concentration-dependent material and mechanical properties of the electrode relate to the structural stability and durability of the electrode. However, the techniques that can be used to test those parameters directly are lacking. Therefore, some techniques are explored to measure them indirectly with the development of theoretical models. Based on the multi-beam optical sensor technique, Sethuraman et al. converted the measured curvature of the electrode to the in-plane stress and biaxial modulus of the film by using Stoney’s equation [31,32]. Xie et al. used an in situ optical system for the deformation of the thin film composite graphite and the thin film composite silicon electrodes during the electrochemical process to characterize the relationship between the elastic modulus of the electrode and the Li concentration [33,34]. Generally, for commercial batteries, the active composite layer is much thicker than that of the current collector. Therefore, large bending deformations of the electrode will be induced during the electrochemical cycle. Li et al. analyzed the relationship between the curvature and material properties of commercial electrodes based on the bending model, indicating that the partial molar volume and elastic modulus change nonlinearly with Li concentration [35,36]; however, the elastic modulus of graphite composite electrodes shown in those works has relatively different values [33,35]. This can be attributed to the different components and structures of the active composite layers. Meanwhile, these results all rely on the basic assumptions in the analysis of theoretical model greatly. In particular, when the bending model of bilayer beam electrodes is used to measure Li concentration-dependent material properties, it needs to satisfy the assumption that the Li is uniformly distributed along the length and thickness of the curved electrode. More importantly, improved experiments and methods need to be explored to better satisfy the basic assumptions and improve the reliability of measuring Li concentration-dependent material properties.

In this paper, an optical electrochemical cell with a transparent quartz window was designed which allowed in situ recording of the bending deformation of the electrodes during electrochemical processes. A sufficient distance was set between the graphite composite electrode and the counter electrode, aiming to make a uniform Li distribution along the length of the curved electrode. The bending curvature of four electrodes with different thickness ratios are measured in real-time during galvanostatic electrochemical cycling. Combined with the theoretical model of bilayer beam electrodes, the partial molar volume and the elastic modulus of each electrode are simultaneously quantified three times. This can help check the consistency and judge the uniformity of Li in the active composite layer. Furthermore, the results of the partial molar volume and the elastic modulus were directly used to measure the DIS of four electrodes with different thicknesses during electrochemical cycling. This improved experiment and method could satisfy the basic assumptions of the theoretical model well. In addition, it can improve the reliability of the measurement results, which can be used to evaluate the mechanical properties in commercial battery electrodes during the electrochemical process.

## 2. Bilayer Electrode Based Measurement Method for Lithium Concentration-Dependent Material Properties

**Figure 1:**
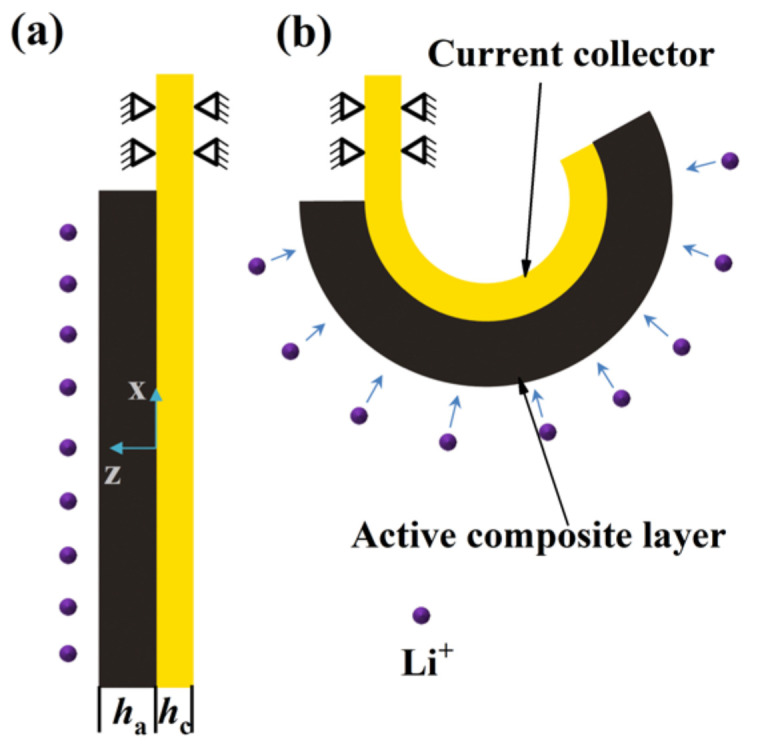
Schematic illustrations of the state of the bilayer beam electrode: (**a**) non-lithiated; (**b**) lithiated.

The lithium concentration-dependent material properties can be measured based upon the bilayer electrode shown in the Figure 1 [12,14,33,34,35,36]. The volume expansion/contraction caused by the intercalation/extraction of Li ions into/from the active composite layer results in bending deformation of the bilayer beam electrode. The bilayer beam electrode becomes a partial circle at the deformed state. According to the theoretical model [14], the bending strain $\varepsilon_{0}$ at the interface between the current collector and the active composite layer and the bending curvature κ are related to the Li concentration as follows:

(1) $$\varepsilon_{0}=\frac{({m_{E}}^{2}{n_{h}}^{4}+3m_{E}{n_{h}}^{2}+4m_{E}n_{h})\Omega c}{3(1+4m_{E}n_{h}+6m_{E}{n_{h}}^{2}+4m_{E}{n_{h}}^{3}+{m_{E}}^{2}{n_{h}}^{4})}$$

(2) $$\kappa =\frac{1}{h_{c}}\frac{2(m_{E}{n_{h}}^{2}+m_{E}n_{h})\Omega c}{1+{n_{h}}^{4}{m_{E}}^{2}+6m_{E}{n_{h}}^{2}+4m_{E}{n_{h}}^{3}+4m_{E}n_{h}}$$

where $m_{E}=E_{a}/E_{c}$ and $n_{h}=h_{a}/h_{c}$ are the elastic modulus ratio and thickness ratio of the active composite layer to the current collector, respectively. Ω and Ωc represent the partial molar volume and swelling strain of the active composite layer, respectively.

In order to obtain the unknown partial molar volume Ω and elastic modulus $E_{a}$ simultaneously, Equation (2) is applied to two bilayer electrodes of different thickness ratios, and two algebraic equations are obtained as follows:

(3a) $$\kappa^{\left(1\right)}{h_{c}}^{\left(1\right)}{n_{h}^{\left(1\right)}}^{4}{m_{E}}^{2}+(P^{\left(1\right)}-2\Omega cn_{h}^{\left(1\right)}-2\Omega c{n_{h}^{\left(1\right)}}^{2})m_{E}+\kappa^{\left(1\right)}{h_{c}}^{\left(1\right)}=0$$

(3b) $$\kappa^{\left(2\right)}{h_{c}}^{\left(2\right)}{n_{h}^{\left(2\right)}}^{4}{m_{E}}^{2}+(P^{\left(2\right)}-2\Omega cn_{h}^{\left(2\right)}-2\Omega c{n_{h}^{\left(2\right)}}^{2})m_{E}+\kappa^{\left(2\right)}{h_{c}}^{\left(2\right)}=0$$

where the superscript (1) and (2) are used to denote the two electrodes of different thickness ratios, and

(4) $$P^{\left(1\right)}=4\kappa^{\left(1\right)}{h_{c}}^{\left(1\right)}n_{h}^{\left(1\right)}+6\kappa^{\left(1\right)}{h_{c}}^{\left(1\right)}{n_{h}^{\left(1\right)}}^{2}+4\kappa^{\left(1\right)}{h_{c}}^{\left(1\right)}{n_{h}^{\left(1\right)}}^{3}$$

(5) $$P^{\left(2\right)}=4\kappa^{\left(2\right)}{h_{c}}^{\left(2\right)}n_{h}^{\left(2\right)}+6\kappa^{\left(2\right)}{h_{c}}^{\left(2\right)}{n_{h}^{\left(2\right)}}^{2}+4\kappa^{\left(2\right)}{h_{c}}^{\left(2\right)}{n_{h}^{\left(2\right)}}^{3}.$$

Solving Equations (3a) and (3b) and the partial molar volume Ω and the elastic modulus $E_{a}$ can be obtained simultaneously as follows:

(6) $$\begin{array}{c} \Omega =\frac{1}{c}\frac{\left(P^{\left(1\right)}\kappa^{\left(2\right)}{h_{c}}^{\left(1\right)}-P^{\left(2\right)}\kappa^{\left(1\right)}{h_{c}}^{\left(2\right)}\right)}{\left((n_{h}^{\left(1\right)}+{n_{h}^{\left(1\right)}}^{2})\kappa^{\left(2\right)}{h_{c}}^{\left(2\right)}-Q\left(n_{h}^{\left(1\right)}+{n_{h}^{\left(1\right)}}^{2}\right)\kappa^{\left(1\right)}{h_{c}}^{\left(1\right)}\right)}+\frac{1}{c}\frac{\kappa^{\left(1\right)}{h_{c}}^{\left(1\right)}\kappa^{\left(2\right)}{h_{c}}^{\left(2\right)}\left({n_{h}^{\left(1\right)}}^{4}-{n_{h}^{\left(2\right)}}^{4}\right)}{2(\left(n_{h}^{\left(1\right)}+{n_{h}^{\left(1\right)}}^{2}\right)\kappa^{\left(2\right)}{h_{c}}^{\left(2\right)}-Q\left(n_{h}^{\left(1\right)}+{n_{h}^{\left(1\right)}}^{2}\right)\kappa^{\left(1\right)}{h_{c}}^{\left(1\right)})}\\ \frac{\left(P^{\left(1\right)}Q-P^{\left(2\right)}\right)+\sqrt{{\left(P^{\left(2\right)}-P^{\left(1\right)}Q\right)}^{2}-\left(\kappa^{\left(2\right)}{h_{c}}^{\left(2\right)}{n_{h}^{\left(2\right)}}^{4}-Q\kappa^{\left(1\right)}{h_{c}}^{\left(1\right)}{n_{h}^{\left(1\right)}}^{4}\right)\left(\kappa^{\left(2\right)}{h_{c}}^{\left(2\right)}-Q\kappa^{\left(1\right)}{h_{c}}^{\left(1\right)}\right)}}{\left(\kappa^{\left(2\right)}{h_{c}}^{\left(2\right)}{n_{h}^{\left(2\right)}}^{4}-Q\kappa^{\left(1\right)}{h_{c}}^{\left(1\right)}{n_{h}^{\left(1\right)}}^{4}\right)} \end{array}$$

(7) $$E_{a}=E_{c}\frac{\left(P^{\left(1\right)}Q-P^{\left(2\right)}\right)+\sqrt{{\left(P^{\left(2\right)}-P^{\left(1\right)}Q\right)}^{2}-\left(\kappa^{\left(2\right)}{h_{c}}^{\left(2\right)}{n_{h}^{\left(2\right)}}^{4}-Q\kappa^{\left(1\right)}{h_{c}}^{\left(1\right)}{n_{h}^{\left(1\right)}}^{4}\right)\left(\kappa^{\left(2\right)}{h_{c}}^{\left(2\right)}-Q\kappa^{\left(1\right)}{h_{c}}^{\left(1\right)}\right)}}{\left(\kappa^{\left(2\right)}{h_{c}}^{\left(2\right)}{n_{h}^{\left(2\right)}}^{4}-Q\kappa^{\left(1\right)}{h_{c}}^{\left(1\right)}{n_{h}^{\left(1\right)}}^{4}\right)}$$

where:

(8) $$Q=\frac{n_{h}^{\left(2\right)}+{n_{h}^{\left(2\right)}}^{2}}{n_{h}^{\left(1\right)}+{n_{h}^{\left(1\right)}}^{2}}.$$

In the experiment, the two bilayer electrodes with different thickness ratios are lithiated and delithiated under the same electrochemical condition, and the curvatures of the two bilayer electrodes are measured and recorded at some given electrochemical states of lithium concentration *c*. Then, the partial molar volume and the elastic modulus can be computed by substituting the measured curvature data into Equations (6) and (7).

It has to be emphasized that Equations (3a)–(7) are derived based upon the assumption that the distribution of lithium concentration *c* is uniform both across the active layer thickness and along the active layer length direction [14]. Although several works have applied low charge rate (about C/10 rate) to ensure a uniform distribution of Li concentration along the thickness of the electrode [33,34,35,36], it is yet to be verified that this assumption is valid in the experiments. Meanwhile, the bilayer beam electrode undergoes a large change in curvature which causes the working bilayer electrode to gradually move away from the counter electrode during electrochemical lithiation. Then the transfer distance of Li ions from the counter electrode to the working electrode gradually increases, which may induce a non-uniform distribution of the lithium concentration along the length of the bilayer electrode [37,38]. Therefore, a key question needs to be addressed: how to improve experiment and method to better satisfy the uniform distribution of lithium throughout the active layer and improve the reliability of measurements?

## 3. Design of Experiments

### 3.1. Improvement of Experiment and Method

**Figure 2:**
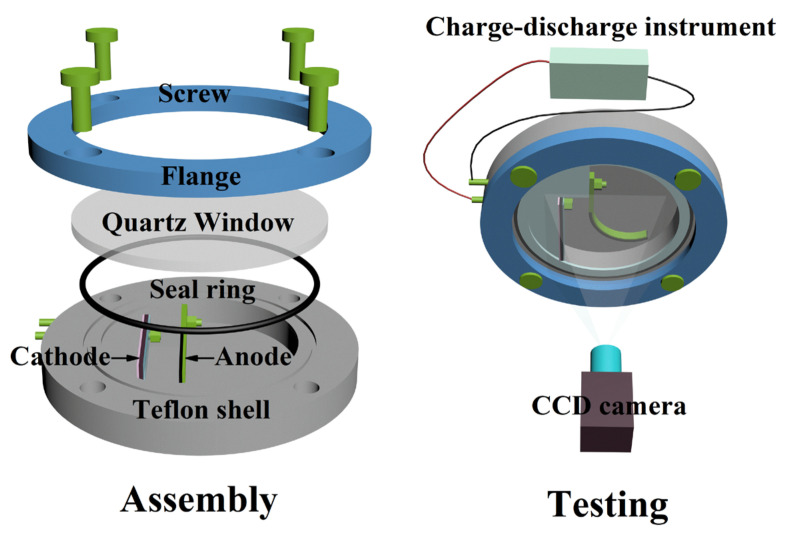
Schematic illustration of in situ optical electrochemical cell assembly and testing.

The distance between the working electrode and the counter electrode is critical to the distribution of lithium concentration [37]. Here, an in situ optical electrochemical cell with a transparent quartz window is designed. The cell allows the adjustment of the distance between the working electrode and the counter electrode for a more uniform distribution of lithium in the active composite layer. The numerical simulation of the curved electrode determined that setting the distance between the working electrode and the counter electrode to approximately 5 mm could be defined as an effective distance to obtain a relatively uniform distribution of Li ions (Supplementary Materials). A schematic illustration of the in situ optical electrochemical cell assembly is shown in Figure 2, and a CCD camera (0.02 mm/pixel resolution) is used to record the bending deformation of the working electrode during the entire electrochemical cycle in real time. The distribution of curvature along the length of bilayer beam electrodes can indirectly determine whether setting a distance of 5 mm between two electrodes satisfies the assumption that the Li concentration is uniform along the length of bilayer beam electrodes.

Graphite composite electrodes are cut into 2 mm × 20 mm cantilever beams as the working electrode, and NCM523 (8 mm × 30 mm, Guangdong Canrd) as the counter electrode to provide ample Li ions. Graphite composite electrodes are charged and discharged with constant current at low current density (ca. C/10) to achieve uniform distribution of Li along the thickness of the active composite layer. The partial molar volume and the elastic modulus of the electrodes, which are intrinsic properties related to the lithium concentration, have no relationship with the thickness of the electrode [14]. Thus, the material’s properties should keep consistent in the electrodes with different thickness under a relatively low charge rate. Therefore, four graphite composite electrodes with different thickness ratios are selected for the experimental measurements, aiming to verify that a low charge rate can keep a uniform distribution along the thickness direction. The detailed parameters of the electrodes are shown in Table 1. Combining Equations (6) and (7), the partial molar volume and the elastic modulus of each electrode are measured several times repeatedly using the same principle. Then the consistency of the measurement results can be used to judge whether the experiments satisfy the basic assumption.

### 3.2. Electrode Preparation and Electrochemical Test

Four graphite composite electrodes with different thickness ratios are fabricated using the following materials: artificial graphite (Particle diameter ~15 μm, theoretical capacity 372 mAhg^−1^), CMC (Sodium carboxymethyl cellulose), and SBR (Styrene butadiene rubber) (Guangdong Canrd). The slurry is mixed by artificial graphite, CMC, and SBR in a weight ratio of 90: 7.5: 2.5. The deionized water is used as solvent. The slurry is mixed using a planetary centrifugal mixer (ARE-310, Thinky). Battery grade copper foil is used as the current collector, and its elastic modulus and yield stress were measured by DMA (dynamic mechanical analysis) tensile test to be 45 GPa and 200 MPa, respectively. The mixed slurry is coated on one side of the current collector. The gap of the blade is adjusted to change the thickness of the active composite layer. The electrodes are dried in a vacuum drying oven (Shanghai Feiyue) at 70 °C for 12 h. Roller press (Shenzhen MTI) is used to improve the conductivity of the electrodes and ensure that all electrodes have a porosity of approximately 31% (See Supplementary Materials for the calculation of the porosity). The thickness of the current collector and active composite layer is measured by Mitutoyo Micrometer (Resolution of 1 µm). 1 M LiPF6 in ethylene carbonate and diethyl carbonate (EC:DEC = 1:1 vol%, Nanjing MJS) is employed as the electrolyte. The in situ optical electrochemical cell is transferred to an argon-filled glove box (MIKROUNA) for assembly. Finally, the assembled cells are taken out of the glove box and rested for 12 h to ensure the electrode was fully infiltrated by the electrolyte.

Electrochemical tests are performed by a charge-discharge instrument (CT-4000-mA NEWARE) at room temperature of 25 °C. All cells are conducted to 5 electrochemical cycles at constant current density (ca. C/10 rate) for 4 h. The upper and lower cut-off voltages are set to 4 V and 2 V, respectively. At the end of each charge and discharge, the open circuit to relax for 5 min. The images of electrode bending deformation are recorded every 30 s by a CCD camera, and electrochemical parameters such as voltage and current are recorded by a charge–discharge instrument.

## 4. Results and Discussion

The diffusion of Li can induce expansion of active particles and the overall expansion of the active composite layer is restricted by the current collector. The mismatch will cause the bending of the bilayer electrode into a partial circle. Here, a transparent quartz window was designed in the electrochemical cell, which allows us to capture the bending deformation of the electrodes real time. The voltage and curvature in the first and second electrochemical cycles were shown in Figure S2. A large amount of Li ions is used to generate the solid electrolyte interface (SEI) in the first electrochemical cycle [39]; thus, the curvature in the second cycle is significantly larger than that of the first one. To avoid the effect of SEI on the estimation of Li concentration, the data after the second electrochemical cycle was chosen for further analysis.

**Figure 3:**
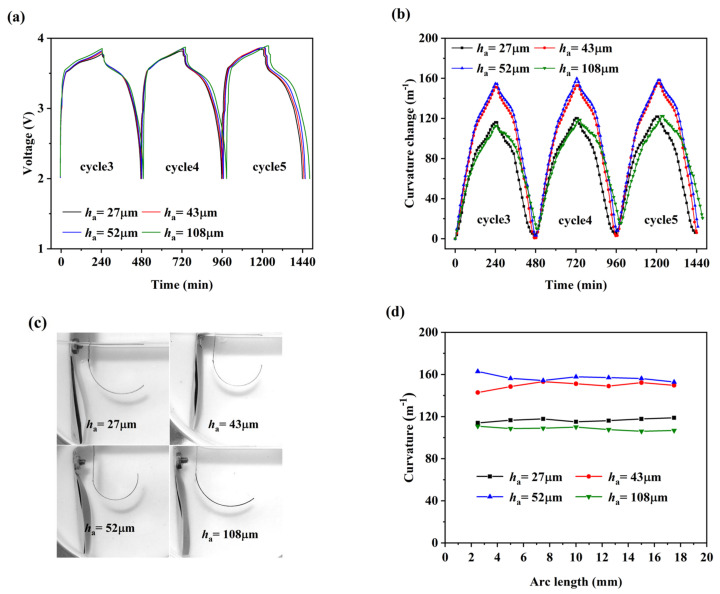
(**a**) Voltage and (**b**) curvature changes in the third, fourth, and fifth electrochemical cycle; (**c**) bending deformation images and (**d**) distribution of curvature along the length direction for four electrodes at the end of the fourth charge.

As shown in Figure 3a, the four cells of different thickness ratios have almost the same voltage profile and the differences between them and are caused by the different coulomb efficiencies, which are over 93% for all cells. Therefore, the optical electrochemical cell can provide an approximate working environment of a coin cell that can maintain a stable reaction process. During the charging process, the bending curvature of the electrode increases greatly with the continuous insertion of Li ions into the active composite layer and the discharging process reverses. Figure 3b shows the highly consistent curvature change of each electrode from the third to fifth electrochemical cycle. Due to the intercalation of Li ions, all graphite composite electrodes undergo a large bending deformation, as shown in Figure 3c. The electrodes with different thickness ratios have different stress states at the same Li concentration, thus causing different deformations [14]. The deformation of the electrodes first increases and then decreases with the thickness increase of the active composite layer. The curvature was measured every 2.5 mm along the length of the electrode; it fluctuated within a small range, indicating that the Li concentration is relatively uniform along the length of the graphite composite electrode, as shown in Figure 3d.

**Figure 4:**
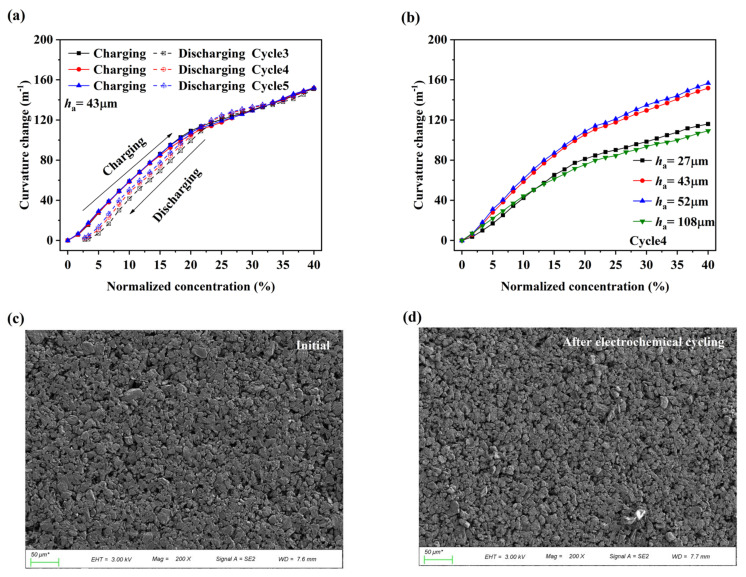
(**a**) Curvature changes of the electrode form the third to fifth cycles, where *h*_1_ = 43 μm; (**b**) curvature changes of the electrode with different thickness ratio at the fourth charging process. Surface morphologies of electrode: (**c**) initial state; (**d**) state after electrochemical cycling.

The electrochemical cells are cycled with a constant current density (ca. C/10 rate) for 4 h. The normalized concentration $\bar{c}$ can be determined by the ratio of the ampere-hour capacity to the maximum theoretical capacity. To further confirm the stability of the electrochemical reaction, the curvature changes correspond to the electrode with the active composite layer thickness of 43 μm is shown in Figure 4a. The slight hysteresis is seen in the curvature evolution during discharging, which may be caused by the asymmetric delithiation and lithiation of graphite materials [40] or the capacity loss caused by the exfoliation of a small amount of graphite particles from the electrode surface.

The curvature data in the fourth electrochemical cycle was used for quantitative analysis, as shown in Figure 4b. The phase transition of graphite in the lithiation process can cause a transformation in its material and structural properties [23,41,42]. The curvature increases nonlinearly with Li concentration, and a transition appears at the normalized concentration of about 18%. This may be related to the phase transition of graphite.

The surface morphologies of the initial and electrochemically cycled graphite composite electrodes were characterized by a scanning electron microscopy (SEM). As shown in Figure 4c,d, the composite active layer is a porous composite material. Compared with the initial electrode, only a small amount of graphite particles was exfoliated from the surface of the electrode after electrochemical cycling, indicating that the graphite composite electrode can maintain the relative stability of the structure even without out-of-plane constraints. The curvature shows a strong consistency across electrochemical cycles, as shown in Figure 4a, indicating that the bending of graphite composite electrodes during the electrochemical process is an elastic behavior.

**Figure 5:**
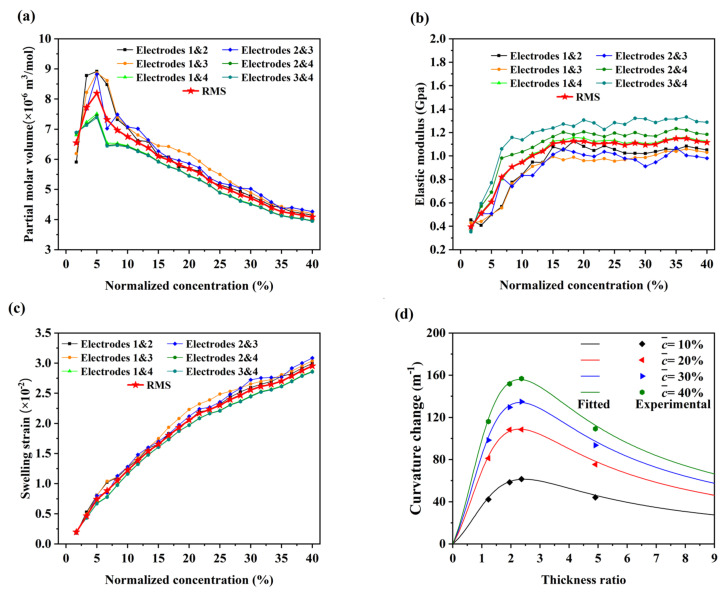
(**a**) The partial molar volume; (**b**) elastic modulus; (**c**) swelling strain of the active composite layer corresponding to normalized concentration; (**d**) the fitted and experimental curvature change corresponding to the thickness ratio at different normalized concentrations.

Based on the measurement method for lithium concentration-dependent material properties of bilayer electrodes, combined with Equations (6) and (7), the partial molar volume and the elastic modulus can be extracted from any two electrodes (an electrode group) with different thickness ratios. We tested the electrodes with four different thickness ratios (Table 1). Each electrode can be combined with the other three electrodes and their partial molar volume and elastic modulus can both be quantified three times, as shown in Figure 5a and Figure 5b, respectively. Due to the accumulation of errors in the manufacture and measurement of electrodes, etc., the extracted partial molar volume and elastic modulus fluctuate in a small range, but the change trend with Li concentration is consistent. It is showed that the graphite composite electrode is charged and discharged at a low rate (ca. C/10 rate) and the concentration gradient of Li along the electrode thickness is small enough, which can ensure the accuracy of the measurement results. As shown in Figure 5a, the partial molar volume with normalized concentration first increases from 6.5 × 10^−6^ m^3^/mol to 8.2 × 10^−6^ m^3^/mol, then decreases almost linearly from 8.2 × 10^−6^ m^3^/mol to 4 × 10^−6^ m^3^/mol. As shown in Figure 5b, the elastic modulus of the active composite layer increases almost linearly from the initial 0.4 GPa; it comes to a steady stage with a value of about 1.1 GPa. The transition of the partial molar volume and the elastic modulus with Li concentration appears different from the curvature, suggesting that the evolution of the curvature is co-determined by the partial molar volume and the elastic modulus. With the lithiation of graphite, the interlayer spacing of graphite increases nonlinearly. In addition, the carbon–carbon bonds within the graphite basal plane are weakened, the interlayer bonds are strengthened, and the polycrystalline Young’s modulus of graphite triples as it is lithiated to LiC6 [23]. The stiffening of the graphite composite electrode with lithiation can be attributed to the stiffening of the graphite. Different with pure graphite, the elastic modulus of the graphite composite electrode changes nonlinearly with lithium concentration and is about one order of magnitude smaller than that of the pure graphite (ca.10 GPa) [23]. This is because the composite electrodes are usually composed of the active particles, conductive carbon black, binder, and pores. It suggests that the partial molar volume and the elastic modulus of the composite active layer is determined by both the graphite and the microstructures of other remaining components. As shown in Figure 5c, the swelling strains have a similar trend with and curvature changes, indicating that the diffusion-induced expansion is the essential factor for the bending deformation of the composite electrodes. Based on the theoretical model, substitute the root mean square (RMS) of the partial molar volume and the elastic modulus into Equation (2), and the fitted curvature with the thickness ratio of the electrodes are shown in Figure 5d. The experimental results are strongly consistent with the fitting results; it further shows that the improved experiment satisfies the basic assumptions in the theoretical model, and that the measurement results are reliable.

**Figure 6:**
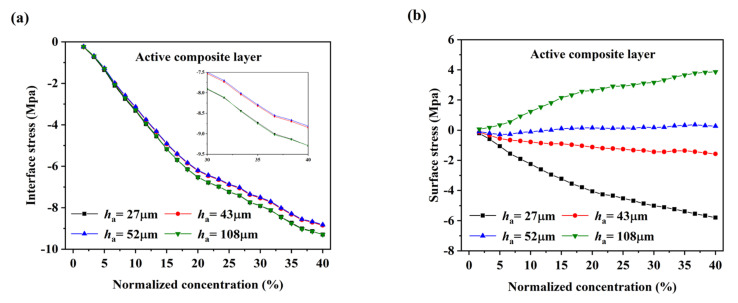

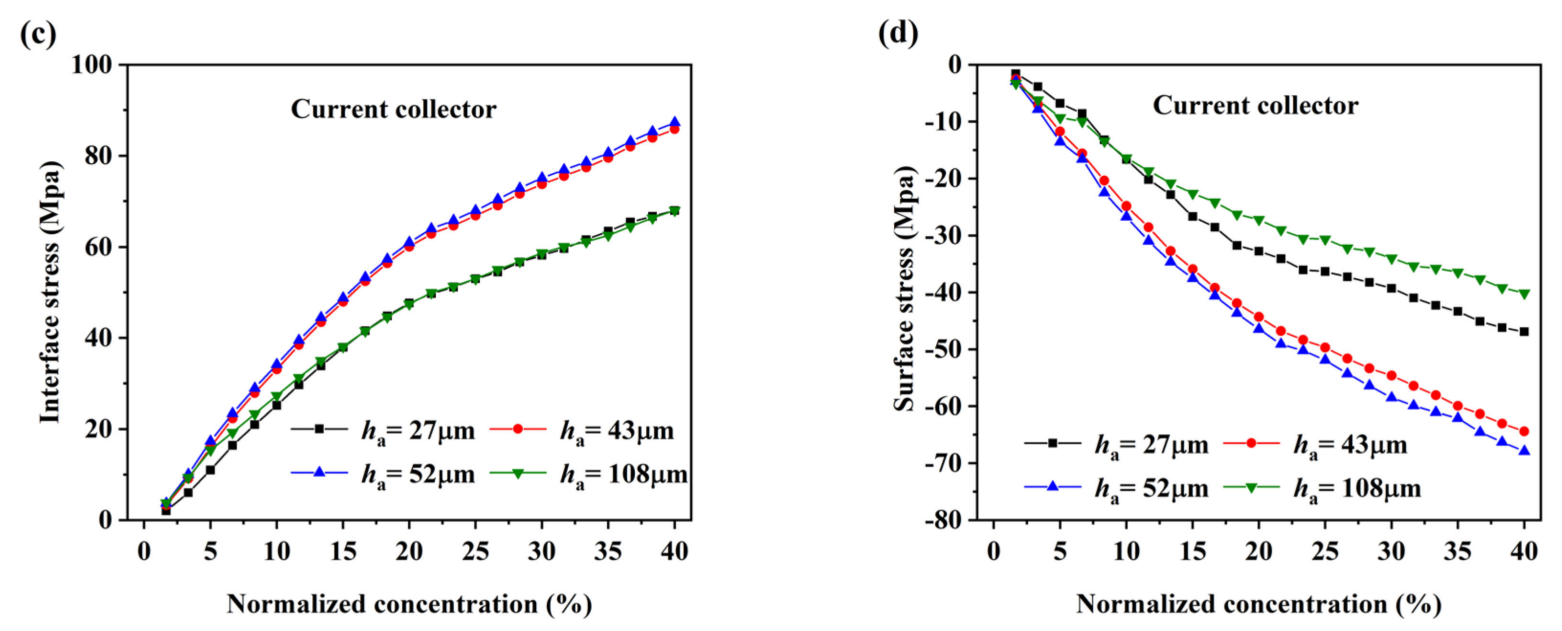
(**a**)The interface stress; (**b**) the surface stress of the active composite layer corresponding to the normalized concentrations; (**c**) the interface stress; (**d**) the surface stress of the current collector corresponding to the normalized concentrations.

The DIS in the electrodes are critical to the durability and structural stability of the battery. The stresses in the active composite layer and the current collector are calculated by substituting the results of measurements of the elastic modulus and swelling strain of graphite composite electrodes into the constructive equations $\sigma_{a}=E_{a}(\varepsilon_{0}+z\kappa -\frac{1}{3}\Omega c)$ and $\sigma_{c}=E_{c}(\varepsilon_{0}+z\kappa )$, respectively [14], as shown in Figure 6. The stress in the electrode increases nonlinearly with increasing Li concentration. The modulus increases in the early stage of lithiation, so the stress increases rapidly at this stage. As the lithiation continues, the elastic modulus hardly changes, while the increase trend of swelling strain gradually becomes smaller, resulting in a slower increase in stress. The interface stress and surface stress of the active composite layer are shown in Figure 6a and Figure 6b, respectively. The active composite layer is subjected to compressive stress at the interface, and first increase and then decrease with the increase of electrode’s thickness ratio, while the surface stress of the active composite layer changes from compressive to tensile with the increase of the electrode’s thickness ratio. The interface stress is the maximum stress in the active composite layer about 10 Mpa, which is about 5% of the stress in the thin film graphite electrode and also follows approximately the same evolutionary trend [43]. The interface stress and surface stress of the current collector are shown in Figure 6c and Figure 6d, respectively. The current collector is subjected to tensile stress at the interface and compressive stress at the surface, respectively. With the increase of the thickness ratio, the stress first increases and then decreases. The maximum stress of the current collector is about 90 Mpa, which is much smaller than the yield stress of the current collector. The compressive stress in the active layer is the main factor to promote the bending deformation of the current collector. As the thickness ratio increases, the surface stress of the active layer changes from compressive to tensile, thus reducing the promoting effect of the active composite layer on the bending deformation of the current collector. This can be used explain why the curvature first increases and then decreases with the thickness ratio.

## 5. Conclusions

In this work, an optical in situ electrochemical cell was designed for recording the bending deformation of graphite composite electrodes with four thickness ratios during electrochemical cycling. Under the large bending deformation of the electrode, it ensures that the Li is uniformly distributed sufficiently along the electrode length. Based on the bending model of bilayer beam electrodes, the partial molar volume and the elastic modulus of the composite electrode were calculated three times. The consistency of the results also indicates that the Li concentration along the electrode thickness direction is sufficiently uniform. The results of curvature measurements and calculations show that the improved experiment satisfies the assumptions in the theoretical model and improves the reliability of measuring the Li concentration-dependent material properties of composite graphite electrodes. In addition, the partial molar volume and the elastic modulus are directly related to the Li concentration and affected by the evolution of the components of the electrode simultaneously; the partial molar volume increases from 6.5 m^3^/mol to 8.2 m^3^/mol and then further decreases to 4.1 m^3^/mol, and the elastic modulus increases nonlinearly from the initial 0.4 GPa to 1.1 GPa as the normalized Li concentration increases. During electrochemical cycling, the mechanical response of the graphite composite electrode is demonstrated to behave in an elastic range. For electrodes with smaller thicknesses ratios, the stress in the active composite layer is compressive stress. As the thickness ratio increases, the surface stress gradually changes from compressive to tensile causing the curvature to first increase and then decrease, while a compressive stress of about 10Mpa is generated at the interface. This work provides a more reliable technique and method for measuring the material and mechanical properties of commercial electrodes during electrochemical cycling. The results deepen our understanding of the working mechanism of LIBs and will assist in the development of next-generation LIBs with excellent performance.

## Figures and Tables

**Table 1:** Parameters of graphite composite electrodes by measurement and calculation.

Parameter	Description	Value				Measurement Method
	Electrode No.	1	2	3	4	
*h*_a_	Thickness of active composite layer	27 μm	43 μm	52 μm	108 μm	Mitutoyo Micrometer
*h*_c_	Thickness of current collector	22 μm	22 μm	22 μm	22 μm	Mitutoyo Micrometer
*n_h_*	Thickness ratio	1.23	1.95	2.36	4.91	*h*_a_/*h*_c_
*i*	Current density	0.131 mA/cm^2^	0.209 mA/cm^2^	0.257 mA/cm^2^	0.524 mA/cm^2^	Calculated (C/10 rate)

## Data Availability

Experiment datas were under further consideration and will supplied later.

## Supplementary Materials

The following supporting information can be downloaded at: https://www.mdpi.com/article/10.3390/nano12244448/s1, Figure S1: Stress-strain curve of the copper foil of 22μm; Figure S2: Voltage (a) and curvature change (b) in the 1st and 2nd electrochemical cycling; Figure S3: Illustration of the optical electrochemical cell structure; Figure S4: The distribution of Li concentration along the length of the curved electrode: (a) surface, (b) interface; Table S1: title; Table S1: Parameters of the 4 types of circular graphite composite electrodes sheet by measurement; Table S2: Newman battery model equations; Table S3: Parameters employed in simulations for the optical electrochemical cell; Table S4: Curvature change data of the 4 electrodes in the 4th charging. References [44,45,46,47] are cited in the supplementary materials.

## References

[1] Tarascon J.-M., Armand M. (2001). Issues and Challenges Facing Rechargeable Lithium Batteries. Nature.

[2] Scrosati B., Hassounab J., Sun Y.-K. (2011). Lithium-ion batteries. A look into the future. Energy Environ. Sci..

[3] Li M., Lu J., Chen Z., Amine K. (2018). 30 Years of Lithium-Ion Batteries. Adv. Mater..

[4] Zhang A., Wang B., Li G., Wang J., Du K. (2020). Fracture analysis of bi-layer electrode in lithium-ion battery caused by diffusion-induced stress. Eng. Fract. Mech..

[5] Li J., Fang Q., Liu F., Liu Y. (2014). Analytical modeling of dislocation effect on diffusion induced stress in a cylindrical lithium ion battery electrode. J. Power Source.

[6] He Y.-L., Hu H.J., Song Y.-C., Guo Z.-S., Liu C., Zhang J.-Q. (2014). Effects of concentration-dependent elastic modulus on the diffusion of lithium ions and diffusion induced stress in layered battery electrodes. J. Power Source.

[7] Kim S., Wee J., Peters K., Huang H.-Y.S. (2018). Multiphysics Coupling in Lithium-Ion Batteries with Reconstructed Porous Microstructures. J. Phys. Chem. C.

[8] Tokur M., Jin M.Y., Sheldon B.W., Akbulu H. (2020). Stress Bearing Mechanism of Reduced Graphene Oxide in Silicon-Based Composite Anodes for Lithium Ion Batteries. ACS Appl. Mater. Interfaces.

[9] Lu B., Yuan Y., Bao Y., Zhao Y., Song Y., Zhang J. (2022). Mechanics-based design of lithium-ion batteries: A perspective. Phys. Chem. Chem. Phys..

[10] Bucci G., Swamy T., Bishop S., Sheldon B.W., Chiang Y.-M., Carter W.C. (2017). The Effect of Stress on Battery-Electrode Capacity. J. Electrochem. Soc..

[11] Mukhopadhyay A., Sheldon B.W. (2014). Deformation and stress in electrode materials for Li-ion batteries. Prog. Mater. Sci..

[12] Li D., Wang Y. (2020). In-situ measurements of mechanical property and stress evolution of commercial graphite electrode. Mater. Des..

[13] Li J., Zhang Q., Xiao X., Cheng Y.-T., Liang C., Dudney N.J. (2015). Unravelling the Impact of Reaction Paths on Mechanical Degradation of Intercalation Cathodes for Lithium-Ion Batteries. J. Am. Chem. Soc..

[14] Zhang J., Lu B., Song Y., Ji X. (2012). Diffusion induced stress in layered Li-ion battery electrode plates. J. Power Source.

[15] Cheng Y.-T., Verbrugge W.M. (2009). Verbrugge Evolution of stress within a spherical insertion electrode particle under potentiostatic and galvanostatic operation. J. Power Source.

[16] Song Y., Lu B., Ji X., Zhang J. (2012). Diffusion Induced Stresses in Cylindrical Lithium-Ion Batteries: Analytical Solutions and Design Insights. J. Electrochem. Soc..

[17] Zhang H., Yang Y., Ren D., Wang L., He X. (2020). Graphite as anode materials: Fundamental Mechanism, Recent Progress and Advances. Energy Storage Mater..

[18] Mei W., Jiang L., Liang C., Sun J., Wang Q. (2021). Understanding of Li-plating on graphite electrode: Detection, quantification and mechanism revelation. Energy Storage Mater..

[19] Yang J., Li Y., Mijailovic A., Wang G., Xiong J., Mathew K., Lu W., Sheldon B.W., Wu Q. (2022). Gradient Porosity Electrode for Fast Charging Lithium-Ion Batteries. J. Mater. Chem. A.

[20] Pham T.A., Kweon K.E., Samanta A., Ong M.T., Lordi V., Pask J.E. (2020). Intercalation of Lithium into Graphite: Insights from First-Principles Simulations. J. Phys. Chem. C.

[21] Holland J., Bhandari A., Kramer D., Milman V., Hanke F., Skylaris C.-K. (2022). Intercalation of Lithium into Graphite: Insights from First-Principles Simulations. Mater. Adv..

[22] Li D., Zhu G., Liu H., Wang Y. (2022). Diffusion-Induced Stress in Commercial Graphite Electrodes during Multiple Cycles Measured by an In Situ Method. Micromachines.

[23] Qi Y., Guo H., Hector L.G., Timmons A. (2010). Threefold Increase in the Young’s Modulus of Graphite Negative Electrode during Lithium Intercalation. J. Electrochem. Soc..

[24] He H., Huang C., Luo C.-W., Liu J.-J., Chao Z.-S. (2013). Dynamic study of Li intercalation into graphite by in situ high energy synchrotron XRD. Electrochim. Acta.

[25] Xu Z., Shi X., Zhuang X., Wang Z., Sun S., Li K., Zhang T.-Y. (2021). Chemical Strain of Graphite-Based Anode during Lithiation and Delithiation at Various Temperatures. Research.

[26] Miao Z., Li Y., Xiao X., Sun Q., He B., Chen X., Liao Y., Zhang Y., Yuan L., Yan Z. (2022). Direct optical fiber monitor on stress evolution of the sulfur-based cathodes for lithium–sulfur batteries. Energy Environ. Sci..

[27] Song H., Xie H., Xu C., Kang Y., Li C., Zhang Q. (2019). In Situ Measurement of Strain Evolution in the Graphene Electrode during Electrochemical Lithiation and Delithiation. J. Phys. Chem. C.

[28] Yao K.P.C., Okasinski J.S., Kalaga K., Shkrob I.A., Abraham D.P. (2019). Quantifying lithium concentration gradients in the graphite electrode of Li-ion cells using operando energy dispersive X-ray diffraction. Energy Environ. Sci..

[29] Qi Y., Harris S.J. (2010). In Situ Observation of Strains during Lithiation of a Graphite Electrode. J. Electrochem. Soc..

[30] Blanquer L.A., Marchini F., Seitz J.R., Daher N., Bétermier F., Huang J., Gervillié C., Tarascon J.-M. (2022). Optical sensors for operando stress monitoring in lithium-based batteries containing solid-state or liquid electrolytes. Nat. Commun..

[31] Sethuraman V.A., Chon M.J., Shimshak M., Srinivasan V., Guduru P.R. (2010). In situ measurements of stress evolution in silicon thin films during electrochemical lithiation and delithiation. J. Power Source.

[32] Sethuraman V.A., Chon M.J., Shimshak M., Winkle N.V., Guduru P.R. (2010). In situ measurement of biaxial modulus of Si anode for Li-ion batteries. Electrochem. Commun..

[33] Xie H., Song H., Kang Y., Wang J. (2018). In Situ Experimental Measurement of the Mechanical Properties of Carbon-Based Electrodes during the Electrochemical Process. J. Electrochem. Soc..

[34] Xie H., Zhang Q., Song H., Shi B., Kang Y. (2017). Modeling and in situ characterization of lithiation-induced stress in electrodes during the coupled mechano-electro-chemical process. J. Power Source.

[35] Li D., Wang Y., Lu B., Zhang J. (2020). Real-time measurements of electro-mechanical coupled deformation and mechanical properties of commercial graphite electrodes. Carbon.

[36] Li D., Wang Y., Hu J., Lu B., Cheng Y.-T., Zhang J. (2017). In situ measurement of mechanical property and stress evolution in a composite silicon electrode. J. Power Source.

[37] Shi B., Han B., Xie H., Kang Y., Zhang Q. (2021). C-rate related diffusion process of the graphite electrode by in situ experiment and analysis. Electrochim. Acta.

[38] Xie H., Han B., Song H., Li X., Kang Y., Zhang Q. (2021). In-situ measurements of electrochemical stress/strain fields and stress analysis during an electrochemical process. J. Mech. Phys. Solids.

[39] Yu Y., Yang Z., Liu Y., Xie J. (2022). Achieving SEI preformed graphite in flow cell to mitigate initial lithium loss. Carbon.

[40] Yao K.P.C., Okasinski J.S., Kalaga K., Almer J.D., Abraham D.P. (2019). Operando Quantification of (De)Lithiation Behavior of Silicon–Graphite Blended Electrodes for Lithium-Ion Batteries. Adv. Energy Mater..

[41] Whitehead A.H., Edstrtim K., Rao N., Owen J.R. (1996). In situ X-ray diffraction studies of a graphite-based Li-ion battery negative electrode. J. Power Source.

[42] Zheng T., Reimers J.N., Dahn J.R. (1995). Effect of Turbostratic Disorder in Graphitic Carbon Hosts on the Intercalation of Lithium. Phys. Rev. B.

[43] Mukhopadhyay A., Tokranov A., Sena K., Xiao X., Sheldon B.W. (2011). Thin film graphite electrodes with low stress generation during Li-intercalation. Carbon.

[44] Doyle M., Fuller T.F., Newman J. (1993). Modeling of Galvanostatic Charge and Discharge of the Lithium/Polymer/Insertion Cell. J. Electrochem. Soc..

[45] Nie J., Sun S., Song Y., Lu B., Soh A., Zhang J.Q. (2021). Impacts of electrode shape on lithiation performance: The edge effect on lithium intercalation. J. Energy Storage.

[46] Ai W., Kirkaldy N., Jiang Y., Offer G., Wang H., Wu B. (2022). A composite electrode model for lithium-ion batteries with silicon/graphite negative electrodes. J. Power Source.

[47] Chen C.H., Planella F.B., O’Regan K., Gastol D., Widanage W.D., Kendrick E. (2020). Development of experimental techniques for parameterization of multi-scale lithium-ion battery models. J. Electrochem. Soc..

