# Supplementary material for: An Improved Experiment for Measuring Lithium Concentration-Dependent Material Properties of Graphite Composite Electrodes
Source: Nanomaterials (Basel). 2022 Dec 14;12(24):4448. doi: 10.3390/nano12244448 (PMC9783245; doi:10.3390/nano12244448)
Supplement: Supplementary file 1 [file nanomaterials-12-04448-s001.zip › nanomaterials-2074163-supplementary.pdf]

Supporting information

An improved experiment for measuring lithium concentration-dependent material properties of graphite composite electrodes

Huibing Liu[a], Guoxing Zhang [a], Dawei Li[b*], Junqian Zhang [a,c*]

a. Shanghai Institute of Applied Mathematics and Mechanics, School of Mechanics and Engineering Science, Shanghai University, Shanghai 200444, China

b. School of Mechanical Engineering, University of Shanghai for Science and Technology, Shanghai 200093, China

c. Zhejiang Laboratory, Hangzhou 311100, China

1. The porosity and the volume fraction of graphite composite electrode

All electrodes are cut into circular electrode sheets with a diameter of 10 mm and their average mass is weighed. According to the relationship between mass, density and volume, the porosity and the volume fraction of graphite composite electrode are calculated as follows:

$$\varphi = \frac{V_{\mathrm{a}} - V_{\mathrm{CMC}} - V_{\mathrm{SBR}} - V_{\mathrm{Gr}}}{V_{\mathrm{a}}} \times 100\%$$

$$\varepsilon = \frac{V_{\mathrm{Gr}}}{V_{\mathrm{a}}}$$

Table S1. Parameters of the 4 types of circular graphite composite electrodes sheet by measurement.

Parameter	Description	Value				Measurement method
	Electrode No.	1	2	3	4	
h_a	Thickness of active composite layer	27μm	43μm	52μm	108μm	Mitutoyo Micrometer
h_c	Thickness of current collector	22μm	22μm	22μm	22μm	Mitutoyo Micrometer
m_e	Mass of electrode	15.02mg	16.84mg	17.86mg	24.19mg	Sartorius balance
m_c	Mass of current collector	11.95mg	11.95mg	11.95mg	11.95mg	Sartorius balance
m_i	Mass of active composite layer	3.07mg	4.89mg	5.91mg	12.24mg	Calculated
ρ_{CMC}	Density of CMC	1.6mg/mm^3	1.6mg/mm^3	1.6mg/mm^3	1.6mg/mm^3	
ρ_{SBR}	Density of SBR	1.04mg/mm^3	1.04mg/mm^3	1.04mg/mm^3	1.04mg/mm^3	
ρ_{Gr}	Density of graphite	2.2 mg/mm^3	2.2 mg/mm^3	2.2 mg/mm^3	2.2 mg/mm^3	
V_a	Volume of active composite layer	2.120mm^3	3.377mm^3	4.084mm^3	8.482mm^3	Calculated
V_{CMC}	Volume of CMC	0.144mm^3	0.229mm^3	0.277mm^3	0.574mm^3	Calculated
V_{SBR}	Volume of SBR	0.074mm^3	0.118mm^3	0.142mm^3	0.294mm^3	Calculated
V_{Gr}	Volume of graphite	1.256mm^3	2.000mm^3	2.418mm^3	5.007mm^3	Calculated
φ	Porosity	30.5%	30.5%	30.5%	30.7%	Calculated
ε	Volume fraction of graphite	59.2%	59.2%	59.2%	59%	Calculated

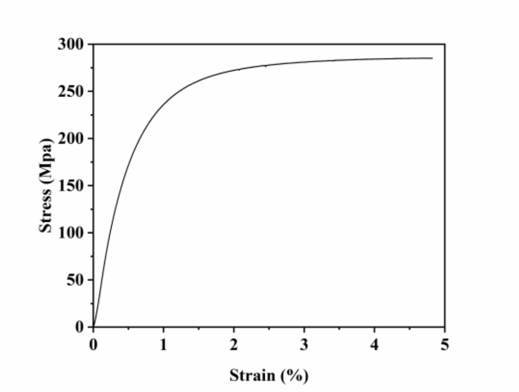

Figure S1 Stress-strain curve of the copper foil of 22μm.

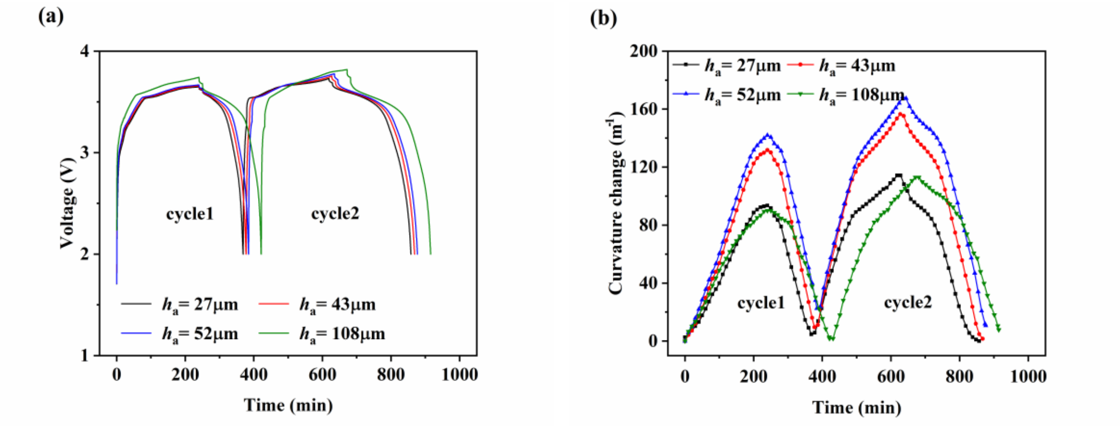

Figure S2. Voltage (a) and curvature change (b) in the 1st and 2nd electrochemical cycling.

2. *Effect of distance on Li concentration distribution*

As shown in Fig. S3, an optical electrochemical cell model is established to determine the effect of the distance ***d*** between the working electrode and the counter electrode (Li metal) on the lithium concentration distribution. The model is based on the Newman battery model [1], which has been widely used for Li-ion batteries and is summarized in Table S2. The parameters of the simulation are summarized in Table S3.

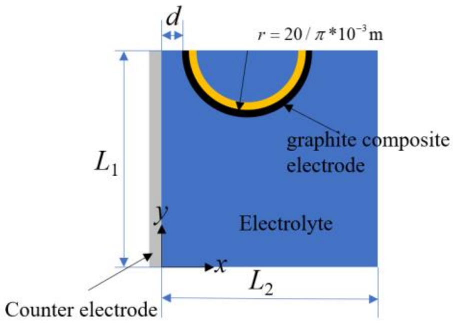

Figure S3. Illustration of the optical electrochemical cell structure.

Table S2. Newman battery model equations [1].

Descriptions	Equations	Boundary Conditions
Diffusion equation, electrolyte phase	$\varepsilon_e \frac{\partial c_e}{\partial t} = \nabla \cdot \left(D_e^{\text{eff}} \nabla c_e\right) + a(1-t_+^0) j_r$	$\nabla c_e \cdot \mathbf{n}_1 = 0$
Diffusion equation, solid phase	$\frac{\partial}{\partial t} c_s - \frac{1}{r^2}\frac{\partial}{\partial r}(r^2 D_s \frac{\partial c_s}{\partial r}) = 0$	$\begin{cases} D_s \frac{\partial c_s}{\partial r}\Big\vert_{r=0} = 0 \\ D_s \frac{\partial c_s}{\partial r}\Big\vert_{r=R_s} = -j_r \end{cases}$
Ohm's Law, electrolyte phase	$\boldsymbol{i}_e = -\sigma_e^{\text{eff}} \nabla \phi_e + \frac{2\sigma_e^{\text{eff}} RT}{F}\left(1+\frac{\partial \ln f}{\partial \ln c_e}\right)\left(1-t_+^0\right)\nabla \ln c_e$	$\nabla \phi_e \cdot \mathbf{n}_1 = 0$
Ohm's Law, solid phase	$\boldsymbol{i}_s = -\sigma_s^{\text{eff}} \nabla \phi_s$	$-\sigma^{\text{eff}} \nabla \phi_s \cdot \mathbf{n}_1 = -i_L$
Butler–Volmer kinetics, Solid-liquid interface	$i = i_0 \left\{ \exp\left(\frac{0.5F}{RT}\eta\right) - \exp\left(\frac{-0.5F}{RT}\eta\right) \right\}$ $i_0 = F k_0 c_e^{1-\alpha} (c_{\max} - c_{\text{surf}})^{1-\alpha} c_{\text{surf}}^{\alpha}$ $\eta = \phi_s - \phi_e - E_{\text{eq}}$	

Table S3. Parameters employed in simulations for the optical electrochemical cell.

Parameter	Description	Value
$\mathbf{n}_1$	Unit normal vector at the interface between the current collector and the active composite layer	
L_1	The length of the cell [mm]	30
L_2	The width of the cell [mm]	30
h_a	the thickness of the active layer [μm]	43
L	the length of graphite composite electrode [mm]	20
r_c	the radius of the curved electrode [mm]	$r_c = L / \pi$
R_s	the diameter of graphite particles [μm]	15
i_L	external current [-2.09A/m^2]	$i_L = \int_L i dL$ $L: y = L_1 - \sqrt{r_c^2 - (x - d - r_c)^2}$
D_s	the diffusivity of lithium in solid phases [m^2/s]	$1.4523 \times 10^{-13} \cdot \exp\left[68025.7 / 8.314 \cdot \left(\frac{1}{318} - \frac{1}{T}\right)\right]$ [2]
ε	the volume fraction of solid phase [%]	59.2
$c_{s,0}$	the initial concentration in solid phase [mol/m^3]	1000
$c_{s,\max}$	the maximum concentration in the solid phase [mol/m^3]	31507
σ_s^{eff}	the electric conductivity in solid electrode [S/m]	100 [2]
E_{eq}	the electrode potential at equilibrium state [V]	$f_1(c) + f_2(c)(T - 298)$ [2]
a	the surface to volume ratio of graphite [1/m]	$a = 3\varepsilon_e / R_s$
D_e^{eff}	the diffusivity of lithium in liquid electrolyte [m^2/s]	$f_3(c) \cdot \exp\left[16500 / 8.314(\frac{1}{298} - \frac{1}{T})\right]$ [2]
ε_e	the volume fraction of liquid electrolyte in graphite composite electrode [%]	30.5
$c_{e,0}$	the initial concentration in liquid electrolyte [mol/m^3]	1000

σ_e^{eff}	the liquid electrolyte conductivity [S/m]	$f_4(c)\cdot\exp\left[4000/8.314(\frac{1}{298}-\frac{1}{T})\right]$ [2]
f	the activity coefficient	$f_5(c)\cdot\exp\left[-1000/8.314(-\frac{1}{T})\right]$ [2]
t_+^0	the transference number	0.2594 [3]
k_0	the reaction rate constant [m/s]	2.123e-10 [4]
T	Temperature [K]	298
$f_i(c)$	the interpolation function	

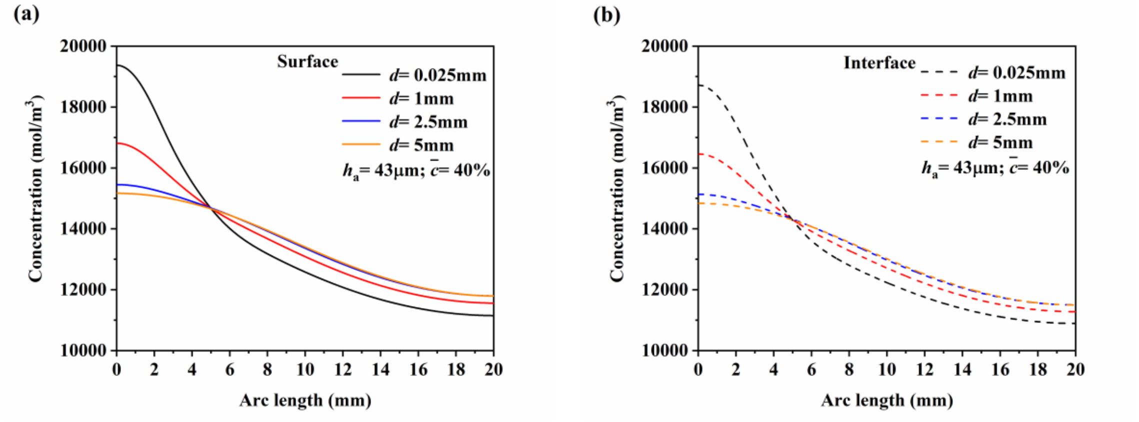

Figure S4. The distribution of Li concentration along the length of the curved electrode: (a) surface, (b) interface.

As shown in Fig. S4, the simulation results show that the lithium concentration decreases along the length of the curved electrode. When the distance between the working electrode and the counter electrode is increased, the lithium concentration gradient along the length direction of the curved electrode gradually decreases. Therefore, the distance between the two electrodes can be adjusted to make the distribution of lithium in the active composite layer more uniform.

Table S4. Curvature change data of the 4 electrodes in the 4th charging.

	Curvature change (m^{-1})			
Normalized concentration (%)	Electrode 1	Electrode 2	Electrode 3	Electrode 4
0.00	0.00	0.00	0.00	0.00
1.67	3.68	5.85	6.72	7.12
3.33	9.99	16.24	18.09	15.73
5.00	17.98	28.06	31.23	24.96
6.67	25.26	38.33	40.41	29.53
8.33	34.54	48.74	51.95	37.14
10.00	42.29	58.48	61.50	44.17
11.67	50.41	67.66	71.16	50.45
13.33	57.28	76.94	79.93	56.41
15.00	65.22	84.71	87.19	61.32
16.67	70.83	92.59	94.85	66.29
18.33	77.38	99.33	102.00	71.54
20.00	81.23	105.40	108.53	75.39
21.67	84.68	110.82	114.28	79.72
23.33	88.12	114.20	117.25	82.66
25.00	90.19	117.77	121.14	84.48
26.67	92.69	121.97	126.01	88.10
28.33	95.89	126.24	130.58	90.46
30.00	98.43	129.65	135.00	93.60
31.67	101.62	133.30	138.23	96.37
33.33	104.90	136.87	141.09	97.88
35.00	107.89	140.89	144.17	99.99
36.67	111.68	144.84	149.21	103.18
38.33	114.04	148.50	153.13	106.62
40.00	116.11	151.76	156.76	109.24

References

[1]. M. Doyle, T. F. Fuller, and J. Newman, "Modeling of Galvanostatic Charge and Discharge of the Lithium/Polymer/Insertion Cell." *Journal of The Electrochemical Society*, **140**, 1526 (1993)

[2]. J. Nie, S. Sun, Y. Song, B. Lu, A. Soh, and J.Q. Zhang, "Impacts of electrode shape on lithiation performance: the edge effect on lithium intercalation." *Journal of Energy Storage*, **47**, 103568 (2021)

[3]. W. Ai, N. Kirkaldy, Y. Jiang , G. Offer, H. Wang, and B. Wu, "A composite electrode model for lithium-ion batteries with silicon/graphite negative electrodes." *Journal of Power Sources*, **527**, 231142 (2022)

[4]. C.H. Chen, F. Brosa Planella, K. O’Regan, D. Gastol, W.D. Widanage, and E. Kendrick, “Development of experimental techniques for parameterization of multi-scale lithium-ion battery models.” *Journal of The Electrochemical Society*, **167**, 080534 (2020)
